# Whole chromosome painting of B chromosomes of the red-eye tetra *Moenkhausia sanctaefilomenae* (Teleostei, Characidae)

**DOI:** 10.3897/CompCytogen.v9i4.5460

**Published:** 2015-10-07

**Authors:** Patricia Elda Sobrinho Scudeler, Débora Diniz, Adriane Pinto Wasko, Claudio Oliveira, Fausto Foresti

**Affiliations:** 1 Universidade Estadual Paulista (UNESP), Instituto de Biociências de Botucatu, Laboratório de Biologia e Genética de Peixes, Departamento de Morfologia, Distrito de Rubião Junior, 18618-970, Botucatu, São Paulo, Brazil; 2 Universidade Estadual do Sudoeste da Bahia (UESB), Departamento de Ciências Biológicas (DCB), Rua José Moreira Sobrinho s/n, 45206-190, Jequié, Bahia, Brazil; 3 Universidade Estadual Paulista (UNESP), Instituto de Biociências de Botucatu, Departamento de Genética, Distrito de Rubião Junior, 18618-970, Botucatu, São Paulo, Brazil

**Keywords:** Chromosomal evolution, Chromosome microdissection, Chromosome painting, Genome organization, Repetitive DNA

## Abstract

B chromosomes are dispensable genomic elements found in different groups of animals and plants. In the present study, a whole chromosome probe was generated from a specific heterochromatic B chromosome occurring in cells of the characidae fish *Moenkhausia sanctaefilomenae* (Steindachner, 1907). The chromosome painting probes were used in fluorescence *in situ* hybridization (FISH) experiments for the assessment of metaphase chromosomes obtained from individuals from three populations of *Moenkhausia sanctaefilomenae*. The results revealed that DNA sequences were shared between a specific B chromosome and many chromosomes of the A complement in all populations analyzed, suggesting a possible intra-specific origin of these B chromosomes. However, no hybridization signals were observed in other B chromosomes found in the same individuals, implying a possible independent origin of B chromosome variants in this species. FISH experiments using 18S rDNA probes revealed the presence of non-active ribosomal genes in some B chromosomes and in some chromosomes of the A complement, suggesting that at least two types of B chromosomes had an independent origin. The role of heterochromatic segments and ribosomal sequences in the origin of B chromosomes were discussed.

## Introduction

Supernumerary or B chromosomes are defined as nonhomologous extra genomic elements that actively recombine with chromosomes of the A complement (Jones and Rees 1982, Camacho et al. 2000). They are also characterized by their peculiar evolutionary mode in the genome of carrier species (Jones and Rees 1982, Beukeboom 1994). Additionally, B chromosomes represent dispensable genetic material and can persist in natural populations without being eliminated by natural selection (Carvalho et al. 2008). Studies on the biology of supernumerary chromosomes include analyses of their distribution among species, structure and origin, inheritance, population dynamics and evolution. They further comprise the integration of B chromosomes with chromosomes of the A complement (Camacho et al. 2000, Bugrov et al. 2007). The occurrence of B chromosomes in fish is uncommon and has been described in about 5% of species already karyotyped (Oliveira et al. 2009, Arai 2011).

The red-eye tetra *Moenkhausia sanctaefilomenae* (Steindachner, 1907) provides a good model for studying B chromosomes in fishes. This species possesses (i) from 0 to 8 B chromosomes (with numbers varying intra- and inter-individually); (ii) B chromosomes with a remarkable polymorphism, morphology and structure (as evidenced by C-banding, with either euchromatic or partially or fully heterochromatic Bs); (iii) putative sex-related B chromosomes (Foresti et al. 1989, Portela-Castro et al. 2001, Hashimoto et al. 2012). However, considering the scarce data about the molecular structure, homology among different B chromosomes and inferences about the origin of *Moenkhausia sanctaefilomenae*, the aim of this work was to apply physical mapping of ribosomal DNA, microdissection and chromosome painting techniques to address these questions.

## Material and methods

We collected 30 individuals (21 males and 9 females) of *Moenkhausia sanctaefilomenae* from Araquá stream 22°44.83'S and 48°28.5'W (DDM), 19 individuals (10 males and 9 females) from Mané-Teixeira stream 22°45.78'S and 48°15.71'W (DDM) and 6 individuals (3 males and 3 females) from Olaria stream 21°9.18'S and 50°3.06'W (DDM), all tributaries of the Tietê River, São Paulo, Brazil. Vouchers were deposited in the fish collection of Laboratório de Biologia e Genética de Peixes, UNESP, Botucatu (LBP 18986/18987/18988, LBP18983 and LBP18982, respectively). The samples were collected in accordance with the Brazilian environmental protection legislation (collection permission MMA/IBAMA/SISBIO - No 3245). The procedures for sampling, maintenance and analysis of the specimens were performed in compliance with the Brazilian College of Animal Experimentation (COBEA) and approved by Bioscience Institute/UNESP Ethics Committee on use of animals (CEUA) (protocol 405).

The mitotic chromosomes were obtained from kidney and gill tissues using the technique described by Foresti et al. (1993). Triple fluorochrome staining with CMA_3_/DA/DAPI followed the procedures described by Schweizer (1980). For DNA extraction, we used the Wizard Genomic DNA Purification Kit (Promega) following the manufacturer’s instructions. Probes of 18S rDNA were obtained by PCR (Polymerase Chain Reaction) from the total DNA of *Moenkhausia sanctaefilomenae* from the Araquá stream using the primers 18S F (5'CCG CTT TGG TGA CTC TTG AT 3’) and 18S R (5'CCG AGG ACC TCA CTA AAC CA 3’) (White et al. 1990).

Microdissection of the B chromosome which are easily identified after GC-specific triple fluorochrome staining (CMA_3_/DA/DAPI), was performed using an Eppendorf Transfer Man NK2 micromanipulator coupled to a Zeiss Axiovert 100 microscope. Ten B chromosomes, previously identified as heterochromatic B chromosomes using the CMA_3_/DA/DAPI staining protocol, were microdissected and transferred into a microcentrifuge tube containing a DOP-PCR mix solution. The probes, here referred to as MsB, were obtained by PCR (Polymerase Chain Reaction) using a DOP primers according to Telenius et al. (1992) and the technique described by Diniz et al. (2008).

For FISH experiments, the chromosomes were treated according to the procedures described by Pinkel et al. (1986). The probes were labeled by PCR with biotin-16-dUTP (Roche Applied Science) and the signal was detected with avidin-FITC (Roche Applied Science). Or else, they were labeled with Digoxigenin-11-dUTP (Roche Applied Science) and the signal was detected with anti-digoxigenin-rhodamine (Roche Applied Science). The images were captured with a digital camera (Olympus DP70) attached to an Olympus BX61 epifluorescent photomicroscope. Image treatment, including karyotype mounting and optimization of brightness and contrast, was performed using the Adobe Photoshop CS2 program.

## Results

**Figure 1.**
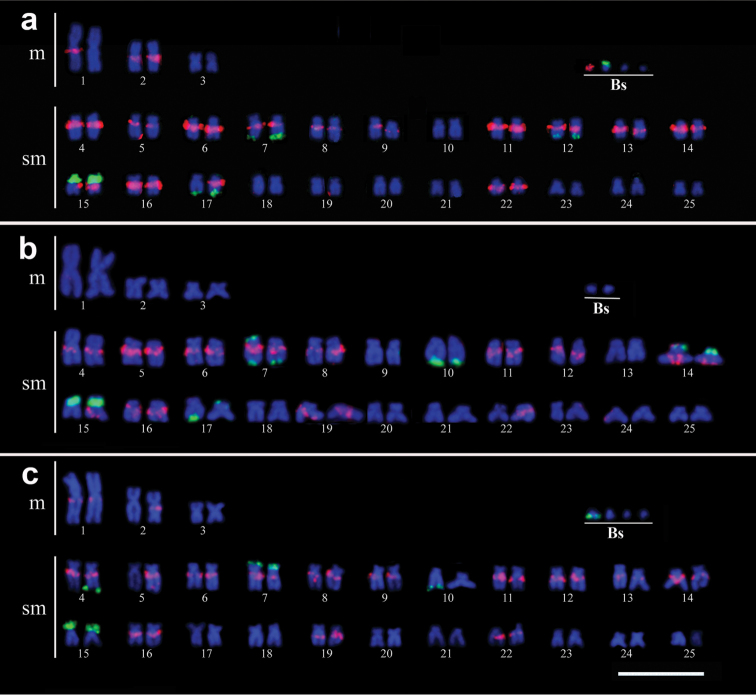
Karyotypes of *Moenkhausia sanctaefilomenae* from the Araquá (**a**), Mané Teixeira (**b**) and Olaria (**c**) streams, arranged from chromosomes after double-FISH. Chromosome painting with MsB-probe (red) and 18S rDNA (green). Bar =10 µm.

Hybridization with the MsB probe on metaphase chromosomes of individuals of the Araquá stream resulted in the complete painting of one B chromosome (Fig. 1a), present in 17% of all specimens analyzed. Additionally, fluorescent signals were observed in the pericentromeric region of 16 chromosome pairs in the A complement. Nevertheless, in some cases a single chromosome of a pair was stained as observed in pair 1 (Fig. 1a). FISH experiments with 18S rDNA probes showed positive signals in pairs 7, 12, 15 and 17 of the A complement and in one B chromosome (Fig. 1a), present in 100% of all specimens analyzed.

Individuals from the Mané Teixeira stream showed hybridization signals in the pericentromeric region of 12 chromosome pairs in the A complement and hybridization signals in only one of the homologous of pairs 15 and 22 with the MsB probe. Signals with the 18S rDNA probe were identified in pairs 7, 10, 14, 15 and 17 (Fig. 1b). In pair 7, one chromosome occured with positive signals in the terminal position of both short and long arms. In pair 17 one chromosome appeared with positive signals in the terminal position of the long arm and the putative homologous with positive signals in the terminal position of the short arm (Fig. 1b). In this population, the MsB and 18S probes did not hybridize with the B chromosomes (Fig. 1b).

Hybridization with the MsB probe in the individuals collected in the Olaria stream showed signals in the pericentromeric region of chromosome pair 15 in the A complement. Notwithstanding, in some cases, a single chromosome of a pair was stained, as observed in pairs 2 and 5 in (Fig. 1c). No hybridization signal was observed in the B chromosomes. FISH with 18S rDNA probes showed positive signals in pairs 4, 7, 10 and 15, but only one of the chromosomes of pairs 4 and 10 were stained (Fig. 1c). B chromosome with 18S sequences were observed in 100% of all specimens analyzed (Fig. 1c).

## Discussion

The B chromosomes observed in the populations of *Moenkhausia sanctaefilomenae* were all very small and characterized as B microchromosomes by Foresti et al. (1989). The occurrence of inter- and intra-individual variation was observed in relation to the number of B chromosomes in the cells of individuals carrying as many as six Bs. Such variation is with accordance with the study by Foresti et al. (1989), who also examined a population of the Tietê River Basin. On the other hand, the individuals from the Paraná River studied by Portela-Castro et al. (2001) showed karyotype differences due to the presence of 0-2 B chromosomes, which were reported to occur only in males. Study by Hashimoto et al. (2012) also showed an inter- and intra-individual variation of the number of B chromosomes in a population from the Batalha River, another tributary of the Tietê River, with individuals carrying up to eight B microchromosomes with a modal number between 2 and 3. All these results showed that the variation in number of B chromosomes in *Moenkhausia sanctaefilomenae* is very high and that different populations have different modal number of B chromosomes.

Our results of chromosome painting with the DNA probes obtained from the B heterochromatic chromosome from the Araquá Stream population (here referred to as MsB) showed that specific hybridization signals were observed in the pericentromeric region of many chromosomes of the A complement and in only one type of B chromosome. In *Moenkhausia sanctaefilomenae*, those pericentromeric regions are heterochromatic (Foresti et al. 1989; present study not showed), the hybridization signals found in most chromosomes of the A complement imply that the microdissected B chromosome may have originated from the heterochromatic region of chromosomes of the A complement, as was also shown by Teruel et al. (2009). This finding suggests an intra-specific origin for this B chromosome. Interestingly, putative homologues of A chromosomes inside populations and in different populations have a slightly different pattern of staining with the MsB probe (Fig. 1). This may be caused by small differences involving the MsB sequences in the A chromosomes occur.

The presence of B chromosomes with no signals of hybridization with the specific B chromosome probe pointed to an independent origin of these elements. Alternatively, it is possible to suppose that: (i) in some B chromosomes, the number of the MsB sequences were too small to be detectable by the technique used here; (ii) the loss of some specific chromosome segments during the dynamic process of modification occurred in the independent evolution of different B chromosome lineages; (iii) the B chromosomes that did not stain with the MsB probe may represent B chromosomes derived from different heterochromatic regions; and (iv) the B chromosomes that did not stain with the MsB probe may represent chromosomes recently originated from euchromatic segments.

Sharing of repetitive DNA sequences between B chromosomes and the elements of the A complement is considered a common feature in the chromosomes of different organisms, such as in the mammals *Vulpes vulpes* (Linnaeus, 1758) (Yang et al. 1999) and *Apodemus peninsulae* (Thomas, 1907) (Rubtsov et al. 2004) and in the insects *Podisma sapporensis* Shiraki, 1910 (Bugrov et al. 2004), *Podisma kanoi* Storozhenko, 1994 (Bugrov et al. 2007) and *Locusta migratoria* Linnaeus, 1758 (Teruel et al. 2009). Among fish, we also have some reports of sequence sharing between A and B complements, as in the case of *Prochilodus lineatus* (Valenciennes, 1836) in, whose SATHI satellite DNA is shared by both B chromosomes and components of the A set (Jesus et al. 2003; Artoni et al. 2006). However, not all heterochromatic segments in *Prochilodus lineatus*, are composed of SATHI satellite DNA, indicating that other families of repetitive DNA could participate in the structure of the chromosomes in this species. The intra-specific origin of B chromosomes of *Astyanax scabripinnis* (Jenyns, 1842) was also corroborated by Vicari et al. (2011), with the use of chromosome painting.

FISH with 18S probes revealed high variability in the *Moenkhausia sanctaefilomenae* samples studied here, involving seven chromosome pairs (Fig. 1). However, the number of active NORs is always three: one chromosome pair (pair 15) and a single chromosome of a pair, as observed by Foresti et al. (1989) for this species. These observations are similar with the results obtained for *Triportheus venezuelensis* Malabarba, 2004 by Nirchio et al. (2007), in which the sequences of 18S rDNA were distributed in nine pairs of chromosomes, but the number of chromosomes with active NORs always remained lower. Studies conductd by Daniel et al. (2012) and Silva et al. (2013) detected up to eight sites of 18S rDNA genes in populations of *Astyanax bockmanni* Vari & Castro, 2007, reinforcing the idea that in this species, sites and location of 18S rDNA are usually multiple and variable.

The presence of ribosomal sites in B chromosomes of *Moenkhausia sanctaefilomenae* suggests that these chromosomes had 18S sequences. Nevertheless, as they are not silver-stained, they may not correspond to active NORs (Foresti et al. 1989, Portela- Castro et al. 2001, Dantas et al. 2007). On the other hand, in fish from the Olaria Stream, ribosomal sites were observed in one B chromosome after silver staining (data not shown here). Mitchell-McGrath and Helgeson (1998), studying hybrids of *Solanum brevidens* Phil. and *Solanum tuberosum* L. and Houben et al. (1997), studying the genus *Brachycome* Cass. suggest that NOR sites are prone to chromosome breakage in plants supporting the hypothesis that B chromosomes could be generated by chromosome fragments coming from these regions. The present results reinforce the hypothesis that chromosome rearrangements involving NOR-bearing chromosomes may be related to the origin of B chromosomes.

According to Lim and Simmons (1994) and Dimitri et al. (1997), the accumulation of repetitive DNA sequences, including transposable elements in specific areas of the chromosomes can render such sites prone to chromosomal rearrangements. Additionally, the molecular structure analysis shows that these B chromosomes may be subject to gene silencing, accumulation of repetitive DNA and also to heterochromatinization processes (Leach et al. 2004). Therefore, the presence of large heterochromatic segments in the A and in some B chromosomes of *Moenkhausia sanctaefilomenae*, and the reduced number of active ribosomal genes in these chromosomes may corroborate the above hypothesis about the gene silencing due to their close relationship with heterochromatin.
